# CbCTB2, an O-methyltransferase is essential for biosynthesis of the phytotoxin cercosporin and infection of sugar beet by *Cercospora beticola*

**DOI:** 10.1186/1471-2229-13-50

**Published:** 2013-03-22

**Authors:** Cornelia Staerkel, Marike J Boenisch, Cathrin Kröger, Jörg Bormann, Wilhelm Schäfer, Dietmar Stahl

**Affiliations:** 1 Department of Molecular Phytopathology and Genetics, University of Hamburg, Biocenter Klein Flottbek, Ohnhorststr. 18, Hamburg, 22609, Germany; 2 KWS SAAT AG, Grimsehlstr. 31, Einbeck, 37555, Germany

## Abstract

**Background:**

Cercospora leaf spot disease, caused by the fungus *Cercospora beticola*, is the most destructive foliar disease of sugar beets (*Beta vulgaris*) worldwide. Cercosporin, a light-inducible toxin, is essential for necrosis of the leaf tissue and development of the typical leaf spots on sugar beet leaves.

**Results:**

In this study we show that the O-methyltransferase gene *CTB2* is essential for cercosporin production and pathogenicity in two *C. beticola* isolates*.* We established a transformation system for *C. beticola* protoplasts, disrupted *CTB2*, and transformed the Δ*ctb2* strains as well as a wild type strain with the DsRed reporter gene. The Δ*ctb2* strains had lost their pigmentation and toxin measurements demonstrated that the Δ*ctb2* strains were defective in cercosporin production. Infection of sugar beets with the wild type and Δ*ctb2* DsRed strains showed that the deletion strain was severely impaired in plant infection. Histological analysis revealed that the *CTB2*-deficient isolate cannot enter the leaf tissue through stomata like the wild type.

**Conclusions:**

Taken together, these observations indicate that cercosporin has a dual function in sugar beet infection: in addition to the well-known role in tissue necrosis, the toxin is required for the early phase of sugar beet infection.

## Background

*Cercospora beticola*, an ascomycete, is the economically most important foliar pathogen of sugar beet (*Beta vulgaris*). Characteristic symptoms of *C. beticola* infection include brown leaf spots extending from the adaxial to the abaxial sides of the leaf; from spring to summer the spots increase in number as the disease progresses, finally coalescing such that the entire leaf turns brown and shrinks. Leaf spot disease is economically critical because the destruction of the leaves limits photosynthesis and compromises yield [1].

*C. beticola* propagates by macroconidia, asexual spores that overwinter in plant debris on the field and are spread in spring by wind and rain splash [2]. *C. beticola* is a hemibiotrophic fungus with biotrophic and necrotrophic phases. Approximately 3-4 days after inoculation, the fungus enters the host through open stomata [3]; after penetration, fungal hyphae grow intercellularly and colonize the leaf parenchymal tissue in an asymptomatic manner [4,5]. The final, necrotrophic phase is characterized by the formation of necrotic lesions, resulting in the leaf spots that are the typical sign of this disease. Unlike other leaf pathogens that necrotize from pinpoint lesions and expand outward, lesions produced by *C. beticola* involve the near-simultaneous collapse of cells in an area many millimeters in diameter. The necrotized tissue becomes the site of conidiophore and conidial development. Although lesions can expand after the initial tissue collapse, the increase in necrotic area on the leaf surface is due primarily to an increase in the number of lesions. One sporulation cycle under field conditions lasts approximately 12 days [1].

The *Cercospora* family has many members that have different hosts, including tobacco, soybean, coffee, rice, corn, and peanut [6]. *Cercospora* species produce cercosporin, a photo-activated toxin that generates reactive oxygen species upon exposure to light. The cercosporin biosynthesis pathway in *C. nicotianae* has been shown to include eight genes, *CTB1*-*CTB8*. *CTB1*, a polyketide synthase, condenses and decarboxylates the precursors malonyl-CoA and acetyl-CoA to form a polyketide. It then catalyzes Claisen condensation and ring closure of the molecule. The next steps are oxidation and hydration reactions executed by *CTB3*, an O-methyltransferase and FAD-dependent monooxygenase, *CTB5*, a FAD/FMN-dependent oxidoreductase, *CTB6*, a NADPH- dependent oxidoreductase, and *CTB7*, another FAD/FMN-dependent oxidoreductase. The subsequent methylation step is carried out by *CTB2*, also an O-methyltransferase, and *CTB3*. The resulting polyketomethylene is dimerized and then exported from the cells by *CTB4*, a major facilitator superfamily transporter. This export mechanism leads to autoimmunity of *C. beticola* against its own toxin. Cercosporin expression is regulated by the zinc-finger transcription factor *CTB8*.

Previous studies have demonstrated that individual inactivation of *CTB1*, *CTB2*, *CTB3*, or *CTB8* leads to the transcriptional inhibition of the entire gene cluster and prevents cercosporin production in *C. nicotianae*[7,8]. The current study aimed to determine whether cercosporin production in *C. beticola* could be reduced by a single knock-out mutation, and to identify differences in the infection behaviors of wild type and toxin-deficient strains.

## Results

### Disruption of *CTB2* in the fungal isolates Ahlburg and Ferrara

Primers were designed using the homologous sequence from *C. nicotianae* (accession number DQ991505). *CTB2* of isolate Ferrara was amplified via PCR; sequencing revealed that the coding region of the *CbCTB2* gene is 91.6% identical to the coding region of *CnCTB2* gene. The derived amino acid sequence of *CbCTB2* is 96% identical to the protein from *C. nicotianae.*

**Figure 1:**
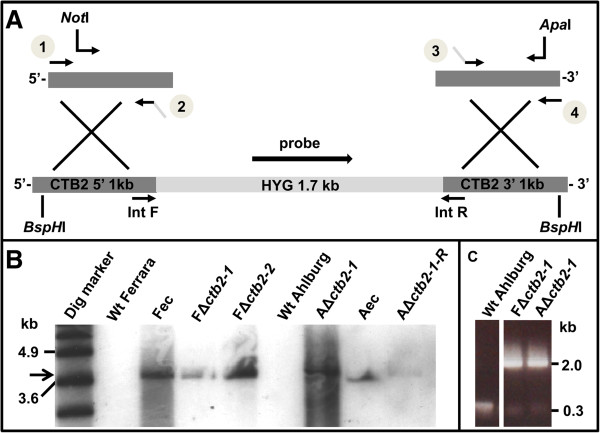
**Disruption of *CTB2 *gene in *C. beticola *isolates Ahlburg and Ferrara. A** The 800 bp 5^′^ and 3^′^ fragments of *CTB2* were amplified separately with primer pairs 1/2 and 3/4, fused with the hygromycin cassette by overlapping regions, and amplified with nested primers (including *Not*I and *Apa*I sites) before cloning into pGEMT. **B** Example of Southern blotting with a hygromycin probe, from left to right, wild type Ferrara (no band), Ferrara ectopic, Ferrara *ctb2* gene disruption strains *2-1* and *2-2*, wild type Ahlburg, Ahlburg *ctb2* disruption strain *2-1*, Ahlburg ectopic, Ahlburg *ctb2* disruption strain *2-2* (band at 3.7 kb, black arrow). Dig VII marker (Roche) was used as DNA size ladder. **C** Confirmative PCR with CTB2 internal primers located adjacent to the integration locus (IntF and IntR), from left to right, wild type Ahlburg (band at 0.3 kb), Ferrara *cbt2* disruption strain *2-1*, Ahlburg disruption strain *2-1* (band at 2 kb) *F*Δ*ctb2-2* and *A*Δ*ctb2-2* produced the same PCR bands as the correspondent disruptants (data not shown).

The gene was disrupted in two isolates of *C. beticola*, Ferrara and Ahlburg. Transformation resulted in 44 Ferrara and 23 Ahlburg transformants. For both isolates, two independent transformants were selected with a disrupted *CTB2* gene (Figure 1). In the Ferrara isolate, *CTB2* gene disruptions (Δ*ctb2*) are subsequently called *F*Δ*ctb2-1* and *F*Δ*ctb2-2* and accordingly in the Ahlburg isolate *A*Δ*ctb2-1* and *A*Δ*ctb2-2*. Ectopic transformants from each wild type were selected and called Fec (Ferrara) and Aec (Ahlburg).

The Ahlburg wild type strain and the deletion mutants *A*Δ*ctb2-1*were selected for transformation with DsRed resulting in more than 20 DsRed fluorescent transformants of each strain. DsRed reporter strains were obtained by transformation of *C. beticola* with pII99DsRed [9], which was linearized with X*ho*I. *CBT2* deletion mutants transformed with DsRed were pink because the red protein was visible in the white hyphae. A DsRed Ahlburg wild type strain expressing DsRed (wt Ahlburg-R) and *A*Δ*ctb2-1* expressing DsRed (*A*Δ*ctb2-1*-*R*) was chosen for further analysis. Growth rates and conidiation of the transformants were not changed.

### Δ*ctb2* transformants are reduced in pigmentation and toxin production

The gene disruption strains lost the characteristic dark pigmentation and appeared white, while the ectopic and wild type colonies were gray. Other properties such as conidiation and growth rate remained unchanged (data not shown).

**Figure 2:**
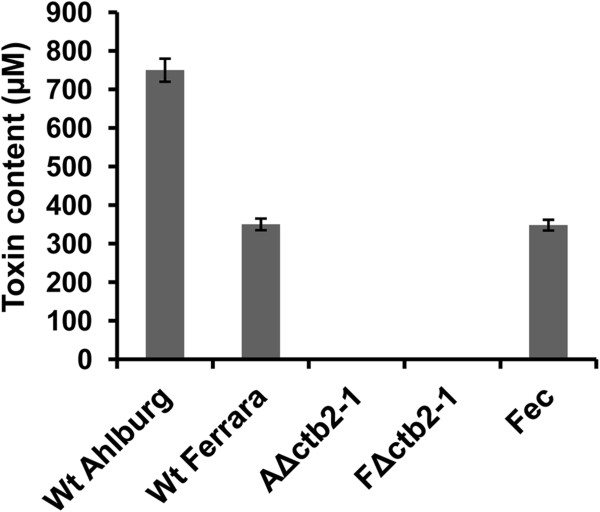
**Quantitation of toxin levels in various isolates.** Toxin production was not detectable for the Ahlburg *cbt2* disruption strain *2-1* and the Ferrara *cbt2* disruption strain *2-1*. Similar results were obtained for mutants *A*Δ*ctb2-2* and *F*Δ*ctb2-2* (data not shown). The more virulent wild type Ahlburg produced 750 μM cercosporin compared to 320 μM cercosporin detected for Ferrara, the less virulent isolate. The ectopic Ferrara mutant produced toxin levels comparable to the Ferrara wild type. Extractions were repeated three times for each isolate. Standard deviations are marked in columns by black lines.

The Ahlburg isolate had a higher toxin content than the Ferrara isolate (Figure 2), which may explain why Ahlburg is the more virulent isolate. The Δ*ctb2* strains from Ahlburg and Ferrara isolates did not produce cercosporin, whereas the toxin content of the ectopic Ferrara transformant was identical to the wild type isolate (Figure 2). Ahlburg ectopic transformant produced similar amounts of cercosporin as the wild type (data not shown).

**Figure 3:**
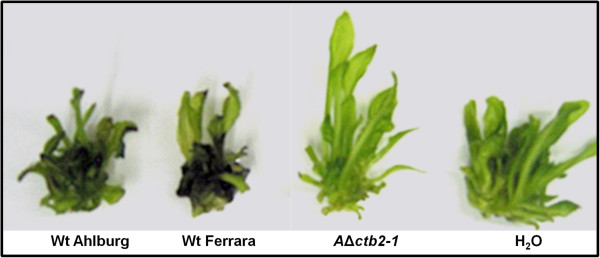
**Sugar beet plants grown *in-vitro *and treated with toxin extracts.** Plants exhibited varying degrees of damage one day after treatment with cercosporin extracts from various fungal isolates and mutants. Plants treated with cercosporin from wild type Ahlburg and Ferrara became necrotic and black (left), but plants treated with cercosporin extract of the Ahlburg disruption strain *2-1* remained green and healthy, comparable with the plant treatment with water which served as the negative control (right). Experiments were repeated three times with five plants for each extract.

When *in vitro*-cultured sugar beets were treated with aqueous solution of cercosporin from PDA plates, the plants dipped in wild type extract died after one day, while plants treated with the water control or the Δ*ctb2* extract remained healthy and green (Figure 3). These experiments were repeated three times with five plants treated in each plate extract. Culture extract from the gene disruption strain therefore lacks a key component needed to damage plant tissue.

### *CTB2* is important for pathogenicity of *C. beticola*

**Figure 4:**
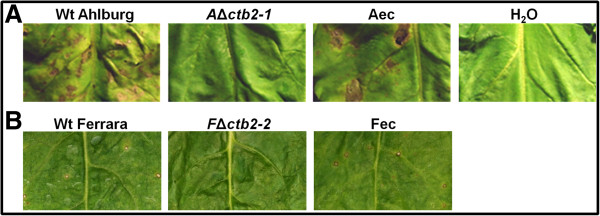
**The Δ*CTB2 *strains fail to cause disease symptoms on sugar beet leaves. A** Enlarged sections of the surface of sugar beet leaves reveal that exposure to the wild type Ahlburg strain led to the characteristic leaf spot symptoms 21 days after inoculation. Ahlburg *ctb2* disruption strain *2-1* failed to cause typical leaf spot symptoms*.* Ahlburg ectopic strain produced wild type like leaf spots. Treatment with water served as the negative control. **B** Ferrara wild type caused leaf spot symptoms, although significantly smaller compared to Ahlburg wild type. Ferrara *cbt2* disruption strain *2-1* failed to cause leaf spot symptoms in contrast to Ferrara ectopic mutant. Experiments were repeated twice with ten plants for each fungal strain. Both disruptants of each wild type isolate gave similar results (data not shown).

Infection of 3-month-old sugar beets with the Ahlburg and Ferrara wild type and the corresponding disruptants *A*Δ*ctb2-1* and *A*Δ*ctb2-2* as well as *F*Δ*ctb2-1* and *F*Δ*ctb2-2* strains demonstrated that the Δ*ctb2* strains of both isolates caused no lesions on the leaves, and the entire plants appeared healthy three weeks after infection. Symptoms caused by the two disruptants of each wild type were indistinguishable (data not shown). Wild type strains of Ahlburg and Ferrara infected the plants and caused lesions. The Ahlburg isolate proved to be highly aggressive causing severe leaf spot lesions comparing to the less virulent Ferrara isolate causing only minor lesions (Figure 4). The symptoms caused by the ectopic transformants of Ferrara and Ahlburg were indistinguishable from the symptoms of the corresponding wild type strain, indicating that *CTB2* is essential for pathogenicity of *C. beticola* (Figure 4).

**Figure 5:**
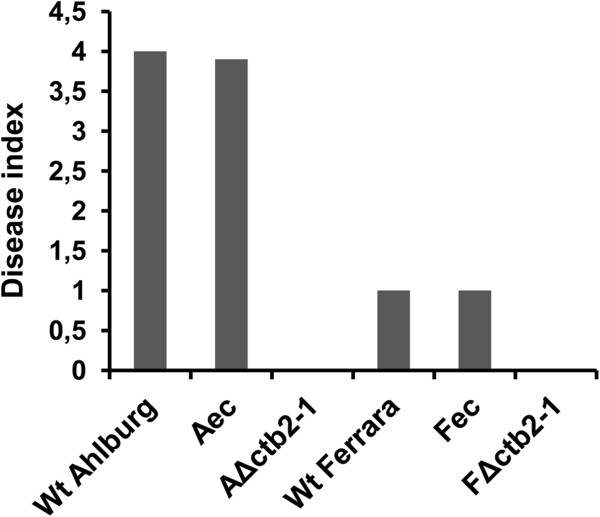
**Disease indices of sugar beets exposed to *C. beticola*.** Plants were infected with conidia of the wild type strains of Ahlburg and Ferrara, with the Ahlburg *ctb2* disruption strain *2-1,* the Ferrara *ctb2* disruption strain *2-1*, and with the ectopic transformants of Ahlburg and Ferrara. The disease severity was rated after 21 days. Plants infected with the Δ*CTB2* strains of both wild types exhibited no symptoms of infection (disease score 0). The wild type strain Ahlburg and the ectopic transformant Ahlburg showed a disease score of 4. The wild type strain Ferrara and the ectopic transformant Ferrara showed a disease score of 1. Δ*ctb2* mutants did not cause lesions.

Disease rating was carried out by assessing the percentage of diseased leaf surface [10]. The disease rating 21 days after infection demonstrated that the plants infected with the gene disruption strain were symptom-free, while the plants infected with the Ahlburg wild type strain had a disease index of 4 on a scale of 9, whereas Ferrara wild type and ectopic strains had a disease index of 1 (Figure 5).

Occasionally, single lesions were observed on some leaves infected with the Ahlburg Δ*ctb2* strains 24 days after inoculation. The application of two different inoculation techniques showed that the appearance of the rare lesions depends on the quality of inoculum. A rough inoculum with clumps of mycelium, which was produced as described [11] favored the appearance of lesions. However, the preparation of a fine spore suspension by filtering and sieving, as described in Material and Methods section, resulted only in very few necrotic spots after 24 days.

### Δ*ctb2* is impaired in penetration of host leaves

**Figure 6:**
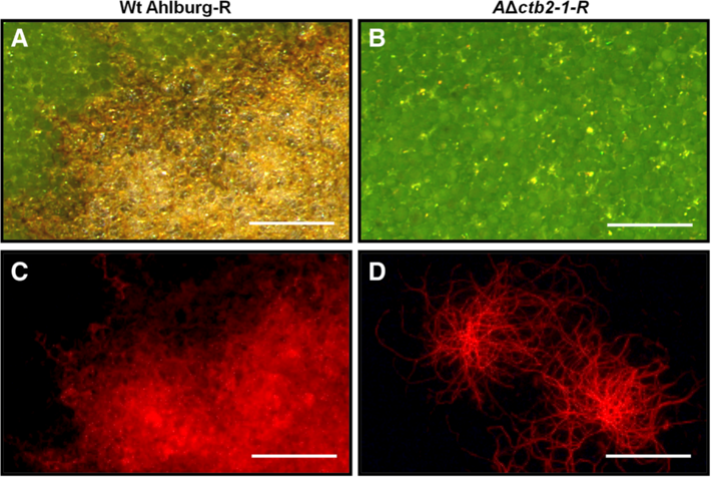
***C. beticola *infection monitored by fluorescence microscopy.** Imaging leaf infection of sugar beet plants with the wild type Ahlburg and the Ahlburg *ctb2* disruption strain *2-1* both transformed to express constitutively DsRed. **A** and **B** Bright field images of the leaf surface, demonstrate characteristic leaf spot symptoms of the wild type infection at 21 days after inoculation, while the gene disruption strain did not cause leaf spots. **C** and **D** The mycelium of DsRed expressing strains of Ahlburg wild type and Ahlburg *ctb2* disruption strain *2-1* growing on the leaf surface was detected with DsRed filterset of Leica MZ FL III microscope. Scale bars: 250 μm.

A histological analysis was performed to evaluate the infection process of the Ahlburg wild type and the Ahlburg Δ*ctb2-1* strain at the cellular level. A constitutive expression of the DsRed fluorescent reporter in Ahlburg wild type and Ahlburg disruptant facilitated the microscopic detection of fungal hyphae. *C. beticola* infection monitored by fluorescence microscopy showed a strong DsRed fluorescence in the necrotic spots caused by the DsRed wild type strain (Figure 6A, C). The Δ*ctb2-1* DsRed strain did not cause leaf spots and the fluorescence was restricted to the hyphae on the leaf surface (Figure 6B, D).

**Figure 7:**
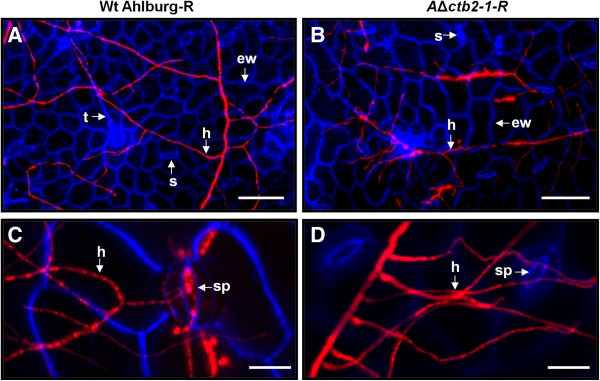
**Colonization and penetration of sugar beet leaves by Ahlburg wild type DsRed and Ahlburg *ctb2 *disruption strain *2-1*-DsRed.** Fluorescence microscopy was performed with a Zeiss fluorescence microscope, using the DsRed filter for detection of the hyphae and UV light to excite blue autofluorescence of the plant cell wall. At 4 days after inoculation, **A** Ahlburg wild type-DsRed mycelia and **B** Ahlburg *ctb2* disruption strain *2-1-DsRed* mycelia were visible on the surface of leaves. **C** At 4 days after inoculation, the wild type approached stomata and began to penetrate. **D** The gene disruption strain grew on the surface without entering through stomata at 4 days after infection. Scale bars: **A** and **B** = 100 μm, **C** and **D** = 20 μm. Abbreviations: ew epidermal cell wall, h hyphae, s stomata, sp stomatal pore, t trichome.

**Figure 8:**
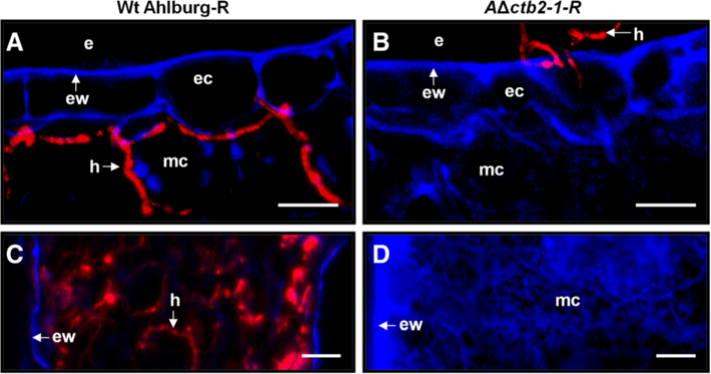
**Invasion of sugar beet leaves by Ahlburg wild type DsRed and Ahlburg *ctb2 *disruption strain *2-1-*DsRed. A** Fluorescence microscopy of leaf cross sections at 21 days after inoculation showed hyphae of the Ahlburg wild type DsRed invading mesophyll cells. **B** Leaf cross sections at 21 days after inoculation with the gene disruption strain did not show invasion of subepidermal layers. The hyphae were not closely attached to the surface and easily dislocated. **C** and **D** 21 days after inoculation the infection was at its peak and symptoms were prominent on the leaves infected with wild type. **C** Cross section through a leaf spot lesion of a leaf infected with wild type. Many hyphae colonize the entire tissue. The loss of blue autofluorescence of the plant cell walls indicates degradation of the host tissue. **D** Cross sections of leaves infected with the gene disruption strain showed strongly increased blue autofluorescence of the plant cell walls, but no hyphal growth. Scale bars: **A** and **B** = 100 μm, **C** and **D** = 20 μm. Abbreviations: e extracellular space, ec epidermis cell, ew epidermal cell wall, h hyphae, mc mesophyll cell.

Fluorescence microscopy of the adaxial and abaxial leaf surfaces showed germination and hyphal spread of the Ahlburg wild type (Figure 7A) and the gene disruption strain (Figure 7B). The wild type strain was beginning to infect through the stomata at 4 days after infection (Figure 7C) while the gene disruption strain did not penetrate the host tissue (Figure 7D). Cross sections of leaves at different stages of the infection revealed that the wild type had progressed intercellularly from the penetration site (Figure 8A) and was beginning to cause necrosis 14 days after infection. The gene disruption strain stopped at the epidermis layer (Figure 8B). The wild type spread massively inside the leaves until 21 days after infection (Figure 8C), when large necrotic lesions became visible on the surface. The gene disruption strain had ceased to grow at this point and failed to spread inside the leaf (Figure 8D).

## Discussion

Despite progress in breeding and fungicide-mediated plant protection, understanding of the molecular interaction between *C. beticola* and sugar beet is just beginning. Previous studies in *C. nicotianae* indicated that the disruption of several genes involved in toxin biosynthesis disabled cercosporin production and rendered the fungus nonpathogenic [12]. In the current study, we showed that the O-methyltransferase gene *CTB2* is essential for cercosporin biosynthesis and fungal virulence of *C. beticola.* Knock-out mutants (Figure 1) failed to produce cercosporin (Figure 2) and were nonpathogenic on sugar beet plants grown in the greenhouse or *in vitro*. The mutant strains lost the characteristic dark pigmentation.

The loss of grey pigmentation of the Δ*ctb2* strains indicates that the disturbance of the cercosporin pathway may affect the formation of fungal pigments. Mycelial pigments like melanin or bikaverin and fusarubins of *Fusarium fujikuroi*[13,14] are synthesized by polyketide synthase gene clusters similar to the Cercosporin biosynthesis gene cluster. Analysis of Δ*ctb2 *mutants of *C. nicotianea* revealed that also transcripts of *CnCTB1, CnCTB3, CnCTB4* and *CnCTB8* are completely down regulated when *CnCTB2* was disrupted [7]. Beside the involvement in cercosporin biosynthesis the CbCTB2 protein or proteins targeted by a CbCTB2 feedback inhibition could be involved in the biosynthesis of other fungal pigments. Beside the essential role for cercosporin biosynthesis the knock-out of the O-methyltransferase gene *CTB2* may therefore have a pleiotropic effect on *C. beticola*. A discoloration of the fungal mycelium was also described for the Δ*ctb1 and* Δ*ctb3* mutants of *C. nicotianae* and explained by the absence of the red-colored cercosporin [15].

Weiland et al. [16] performed site-directed knock-out experiments of *CbCTB1* in *C. beticola*, the polyketide synthase involved in cercosporin biosynthesis. Two analyzed Δ*ctb1* mutants did not produce cercosporin, yet the mutants were able to cause smaller and fewer lesions than the wild type. We observed that the Δ*ctb2* strains also caused rarely necrotic spots, but the frequency of this spots was influenced by the quality of the inoculum. The more the infection technique corresponded to the natural infection condition; the lower was the number of necrosis.

Plant pathogenic fungi must overcome the cell wall in order to enter the host plant, and have therefore developed very efficient and sophisticated mechanisms to breach that barrier. The mode of fungal penetration depends on their lifestyle; *C. beticola* has been shown to enter the host leaves without specific infection structures, invading through the stomata, followed by intercellular growth [4].

Our observations via fluorescence microscopy of reporter strains revealed that the Ahlburg Δ*ctb2* strain is unable to penetrate host tissue and establish an infection. Although the mutant strain germinated well on the surface, maintained growth for several days, and in some cases was able to localize stomata, it failed to grow inside the leaves or to cause disease symptoms, indicating that cercosporin is necessary for tissue colonization during early stages of infection by *C. beticola*. The involvement of cercosporin in the early infection phase of *C. beticola* is unexpected. Earlier studies revealed that purified cercosporin caused necrotic spots on sugar beet leaves that were similar in size and ultrastructural composition to the lesions caused by *C. beticola*[5,17]. Therefore, it was hypothesized that cercosporin’s major contribution was to cause the extensive blighting symptoms characteristic of many Cercospora diseases. After penetration through the stomata and colonization, the membrane-damaging toxin should allow cell breakdown and leakage of nutrients for fungal growth and sporulation [6].

The failure of the *CTB2*-deficient strains to penetrate and colonize leaf tissue raises questions regarding the role of cercosporin in the biotrophic phase of infection. Transcriptome analysis of more than 17.000 sugar beet cDNAs demonstrated that the transcriptional activation of defense reactions in the early to mid stages of *C. beticola* infection is suppressed in polygenic resistant or susceptible genotypes [1]. *C. beticola* has obviously developed a strategy to dampen the immunity triggered by pathogen-associated molecular patterns (PAMP). In the case of the *C. beticola*–sugar beet interaction, the suppression of phenylalanine-ammonium lyase and cinnamate-4-hydroxylase have been described in detail [11]. The repression of phenylalanine-ammonium lyase gene expression was induced by *C. beticola* production of phytohormone abscisic acid during the early phase of infection [18].

It is interesting to speculate that cercosporin may be involved in the suppression of plant defense reactions. As a prerequisite for this model, the toxin must be present in the early phase of infection and easily accessible to the plant cells. Since the biosynthesis of cercosporin is light-regulated [19], it can be assumed that the germinating spores and growing hyphae of *C. beticola* are already producing the toxin on the surface of the epidermis. Furthermore, cercosporin is a lipid-soluble molecule that penetrates rapidly into host cell membranes [6] and can interact with membrane-embedded molecules such as receptors or transporters. However, additional experiments are necessary to demonstrate a direct role of cercosporin in the repression of plant defense mechanisms.

## Conclusions

We disrupted the *CbCTB2* gene coding for an O-methyltransferase thereby eliminating toxin production. The resultant mutants were not only unable to produce the toxin, but failed to infect sugar beet leaves. Cercosporin deficient mutants grow on the leaf surface but were unable to invade and colonize sugar beet leaves. Previously, it was hypothesized that cercosporin is involved in tissue damage after initial penetration and colonization. Here we show that cercosporin plays also a role in the initial biotrophic phase of the pathogen. Recent results showed that *Cercospora beticola* represses transcriptional activation of plant defense pathways. We assume that cercosporin is important for suppressing these defense reactions which are otherwise triggered by pathogen associated molecular patterns. Thereby, cercosporin enables the pathogen to successfully infect its host plant.

## Methods

### Primers and plasmids

Amplification of the hygromycin cassette required primers YgF (5^′^-GTTGGCGACCTCGTATTGG) and HyR (3^′^-CTTACCACCTGCTCATCACCT). The primers CTB2-1F (5^′^-CGCTAGATTTAGGTGTTGGA), CTB2-2R (5^′^-**agatgccgaccgaacaagagctgtccccc**GCAATCTTTCTTCCTATGCT), CTB2-3F (5^′^-**caatgctacatcacccacctcgctccccc**CGTTTCCAAGTCCAAGATCTG), CTB2-4R (5^′^-CTCTTTCGTCCCTCGTATCTC), CTB2-5F (5^′^-AACCTCCTTTGCGTATTCTC), and CTB2-6R (5^′^-ATGTTTCCGAGTTCTTGATGTG) were used for the *CTB2* deletion (hygromycin-overlapping sequences underlined). Finally, primers CTB2-int-F (5^′^-AGCATAGGAAGAAAGATTGC) and CTB2-int-R (5^′^-CAGATCTTGGACTTGGAAACG) served as control primers for the *CTB2* gene. Primers were designed using the homologous sequence from *C. nicotianae* (accession number DQ991505). Plasmids used in this study were pGEMT (Promega, Mannheim, Germany) for TA cloning of PCR fragments, pII99DsRed [9], and pGEMThyg (Le Thi Thu Giang, University of Hamburg, Germany).

### Fungal and bacterial strains

*Escherichia coli* strain XL1-blue (Stratagene, La Jolla, CA, USA) was used for molecular cloning. The *C. beticola* isolates Ahlburg and Ferrara were kindly provided by Planta GmbH, Einbeck, Germany. Ahlburg is known to be the more virulent isolate (unpublished results).

### Media

*C. beticola* was cultured in complete liquid media [20] for DNA extraction, and on complete media agar plates for maintenance. Transformants were grown under selective pressure with 50 μg/ml hygromycin B (Duchefa, Netherlands) and 100 μg/ml geneticin (Invitrogen, Germany), respectively. Toxin production was carried out on potato dextrose agar (PDA) plates (pH 5.6) under ambient light. Bacteria were cultured in Luria-Bertani liquid media or on Luria-Bertani plates with 200 mg/ml ampicillin (Sigma Aldrich, Munich, Germany). MS medium for the *in vitro* culture of sugar beets was prepared as described [21].

### Fusion PCR

*CTB2* was disrupted by double homologous recombination using fusion PCR to generate a construct consisting of a 1219-bp 3^′^ part of the gene, the hygromycin resistance cassette, and a 796-bp 5^′^ part of the gene (Figure 1A). The hygromycin cassette was released from pGEMThyg with *Sma*I, while the 5´- and 3´-parts of CTB2 were amplified from genomic DNA with primers CTB2-1F, CTB2-2R, CTB2-3F, and CTB2-4R. In the fusion PCR, equal amounts of the upper fragment, hygromycin cassette, and lower fragment were fused, and the resulting 3.7-kb fragment was amplified with primers CTB2-nestF and CTB2-nestR. The final PCR product was cloned into pGEMT and released with *Not*I and *Apa*I.

### Fungal transformation

Protoplasts were prepared as described previously [22], and polyethylene glycol-mediated transformation was carried out as previously described [23]. Buffers were prepared as described previously [24]. Selection with the antibiotic hygromycin B (Duchefa, Harlem, Netherlands) was carried out with 50 μg/ml. Retransformation of hygromycin resistant transformants was performed using geneticin (Duchefa, Harlem, Netherlands) selection at a final concentration of 100 μg/ml.

### Southern blotting

Genomic DNA from wild type and gene-disruption strains was digested overnight with *BspHI* (New England Biolabs, Frankfurt am Main, Germany). The digested DNA was separated on a 0.8% agarose gel at 80-100 V. DNA was transferred by capillary blotting to a Hybond NX membrane (Amersham Biosciences, Little Chalfont, UK) and then hybridized with a digoxygenin-labeled (Roche, Mannheim, Germany) DNA hygromycin probe. Detection and visualization procedures were followed according to the manufacturer’s manual (Roche).

### Toxin extraction

Cercosporin was extracted with 5 N KOH as described by Chung [25]. Plates were prepared from PDA at pH 5.6, evenly inoculated with *C. beticola* conidia, and maintained under daylight conditions and room temperature for two weeks. The agar was cut into small squares and covered with KOH overnight in a glass beaker. Agar pieces were removed with a small household sieve. The cercosporin contents of the Δ*CTB2* strains of Ahlburg and Ferrara, as well as the wild type strains and the ectopic transformants were measured after extracting toxin from PDA plates cultured for two weeks in ambient light. Each measurement was repeated three times from three independent extractions. A 5 mM cercosporin standard was kindly obtained from Planta GmbH. Absorbance was measured in a spectrometer (Ultrospec 3000, Pharmacia Biotech) at 480 nm and the cercosporin content was calculated from the molecular weight of 534.51 g/mol (Sigma). For testing on plants, cercosporin was extracted from PDA plates directly with water, which worked as well as extraction with KOH. Plants were dipped in plate extract containing approximately 750 μM cercosporin (isolate Ahlburg) and 320 μM (isolate Ferrara).

### Plant inoculation

Sugar beet plants were grown in a growth chamber at 18°C with 16 hours of light. To produce conidia for the plant inoculation, PDA plates (pH 5.6) were equally seeded with mycelium mashed in a Waring blender and incubated under daylight for two weeks. Roughly one plate was inoculated for each plant to be infected. The conidia were then carefully scraped off the surface with sterile water and a spatula. The scraped material was filtered through a small household sieve, one layer of miracloth, and a 200 μm Wilson sieve to completely remove all agar plucks. The conidia were then counted in a Fuchs-Rosenthal hemocytometer. Twelve-week-old sugar beet plants were inoculated by thoroughly applying 50 ml of a conidia suspension of 2.0*10^4^ conidia/ml onto the adaxial and abaxial leaf surfaces with a spray bottle. Using 50 ml the leaves of one plant were sufficiently covered. Twenty-five plants were inoculated with each fungal strain. The plants were covered with a foil tent for ten days and exposed to 18 hours of light (2.0*10^4^ lux, 400-600 nm = 4.0-6.0*10^3^ K) at temperatures of 24°C for day and 18°C for night.

For testing the toxin on plants grown *in vitro*, one-week-old *in vitro*-cultured plants were dipped into cell-free plate extract or the water control.

### Microscopy

Leaves inoculated with the wild type and Δ*ctb2* strains and strains expressing DsRed were investigated with MZ FL III microscope (Leica Microsystems, Heerbrugg, Switzerland). The microscope was equipped with a Leica 1.0 × objective and Leica DFC 500 fluorescence camera. To visualize plant necroses under white light conditions reflected light of an external halogen lamp KL 1500 Electronic (Schott, Mainz, Germany) was used. The DsRed fluorescence was detected with the Leica DsRed excitation filter at 546/12 nm and a long pass filter at 560 nm. The LAS Leica software (version 2.7.1) was used for image acquisition and procession.

High resolution fluorescence microscopy was performed with Zeiss Axio Imager.Z1 microscope equipped with a Zeiss Apotome and AxioCamMRm CCD camera. A UV (ultra violet) lamp HAL 100 served as light source. DsRed was excited in the range of 538 to 562 nm and detected in the 570 to 640 nm range. The plant apoplast was excited in the range of 335 to 383 nm and its blue autofluorescence was detected at 420 to 470 nm. Image processing, including overlay of independently detected DsRed and plant autofluorescence as well as generation of maximum intensity projections (MIP) of z-stacks were done with Zeiss AxioVision software (version 4.8.1). All presented images are MIPs of the respective z-stack.

### Disease rating

Disease ratings were performed according to a method that relates the amount of leaf spots with disease severity [10]. Single leaves were rated according to the following disease index: absence of necrotic areas (leaf spots) = 0, necrotic area < 1% = 1, necrotic area 2-5% = 2, necrotic area 6-10% = 3, necrotic area 11-20% = 4, necrotic area 21-40% = 5, necrotic area 41-60% = 6, necrotic area 61-80% = 7, necrotic area 81-100% = 8, and leaf dead = 9.

## Competing Interests

The authors declare that they have no competing interests.

## Authors' contributions

CS carried out carried out the molecular genetic studies and analytical assays as well as drafted the manuscript. MJB participated in histology and microscopy as well as in drafting the manuscript. CK carried out the fungal transformation. JB, DS and WS coordinated the experimental work, were involved in analysis and interpretation of data as well as in drafting and revising of the manuscript. All authors read and approved the final manuscript.
